# 3D Printing of Thermo-Responsive Methylcellulose Hydrogels for Cell-Sheet Engineering

**DOI:** 10.3390/ma11040579

**Published:** 2018-04-10

**Authors:** Andrea Cochis, Lorenzo Bonetti, Rita Sorrentino, Nicola Contessi Negrini, Federico Grassi, Massimiliano Leigheb, Lia Rimondini, Silvia Farè

**Affiliations:** 1 Department of Health Science, Università del Piemonte Orientale UPO, Via Solaroli, 17, 28100 Novara, Italy; andrea.cochis@med.uniupo.it (A.C.); rita.sorrentino@med.uniupo.it (R.S.); federico.grassi@med.uniupo.it (F.G.); maxleigheb@libero.it (M.L.); 2 National Interuniversity Consortium of Materials Science and Technology (INSTM), 50121 Florence, Italy; lorenzo.bonetti@polimi.it (L.B.); nicola.contessi@polimi.it (N.C.N.); 3 Department of Chemistry, Materials and Chemical Engineering “G. Natta”, Politecnico di Milano, Piazza Leonardo da Vinci 32, 20133 Milan, Italy; 4 Interdisciplinary Research Center of Autoimmune Diseases IRCAD, Via Solaroli 17, 28100 Novara, Italy

**Keywords:** methylcellulose, thermo-responsive, 3D printing, rheology, cell sheet, endothelial cells

## Abstract

A possible strategy in regenerative medicine is cell-sheet engineering (CSE), i.e., developing smart cell culture surfaces from which to obtain intact cell sheets (CS). The main goal of this study was to develop 3D printing via extrusion-based bioprinting of methylcellulose (MC)-based hydrogels. Hydrogels were prepared by mixing MC powder in saline solutions (Na_2_SO_4_ and PBS). MC-based hydrogels were analyzed to investigate the rheological behavior and thus optimize the printing process parameters. Cells were tested in vitro on ring-shaped printed hydrogels; bulk MC hydrogels were used for comparison. In vitro tests used murine embryonic fibroblasts (NIH/3T3) and endothelial murine cells (MS1), and the resulting cell sheets were characterized analyzing cell viability and immunofluorescence. In terms of CS preparation, 3D printing proved to be an optimal approach to obtain ring-shaped CS. Cell orientation was observed for the ring-shaped CS and was confirmed by the degree of circularity of their nuclei: cell nuclei in ring-shaped CS were more elongated than those in sheets detached from bulk hydrogels. The 3D printing process appears adequate for the preparation of cell sheets of different shapes for the regeneration of complex tissues.

## 1. Introduction

Tissue engineering (TE) is one possible approach in regenerative medicine; its main goal is to overcome limited donor availability and the high risk of rejection, which affects the clinical treatments currently used [1,2,3].

Among possible TE approaches, cell-sheet engineering (CSE) provides an approach that is innovative compared to the use of biodegradable scaffolds or the injection of cell suspensions at the body site, including by a cell carrier. CSE entails developing smart cell culture surfaces, permitting the in vitro culture of cells and the detachment by specific stimuli (i.e., temperature variation) of intact cell sheets (CS), preserving the gap junctions between cells and the extracellular matrix (ECM) proteins [2]. These smart surfaces are usually composed of thermo-responsive polymers, i.e., materials whose chemical-physical properties and rheological behavior changes in response to temperature variations [4,5,6]. Among the possible smart hydrogels used for CSE, methylcellulose (MC), a cellulose derivative obtained by partial substitution of the hydroxyl groups (–OH) with methoxy groups (–OCH_3_), can be mixed in aqueous solution to obtain thermo-responsive hydrogels [7,8,9,10]. On increasing the temperature, MC-based hydrogels undergo a sol-gel transition at the lower critical solution temperature (LCST). Additionally, the sol-gel transition is accompanied by a change in the hydrogel’s water affinity from hydrophilic in the sol state to hydrophobic in the gel state [10].

In the CSE approach, MC-based hydrogels are exploited as substrates to produce CS in vitro. In particular, cells adhere and proliferate on the hydrogel’s hydrophobic surface (in its gel state) at 37 °C (T > LCST). On lowering the temperature (T < LCST), the hydrogel surface turns hydrophilic (in its sol state) and the cell sheets spontaneously detach [7,8]. In addition, the LCST can be varied by adding ionic compounds to the MC aqueous solution. In particular, salting-out and salting-in ions in the MC solution decrease and increase the LCST, respectively [11,12]. As reported elsewhere [13,14], different MC-based hydrogel formulations were prepared and characterized, evidencing two formulations that can be used for the preparation of CS.

Thanks to recent developments in 3D printing technologies, hydrogel 3D printing is now under investigation with the goal of obtaining complex and reproducible shapes of the printed structures. With this approach, hydrogels or cell-laden hydrogels (i.e., bioink) can be printed, layer by layer, to produce engineered constructs [15,16]. The principal 3D bioprinting techniques in the biomedical field are inkjet bioprinting, laser-assisted bioprinting (LAB), and extrusion-based bioprinting (EBB) [17]. Inkjet bioprinting enables hydrogels, or bioinks stored in cartridges, to be printed as drops by means of a nozzle, not in contact with the print platform [15,16,17,18]. LAB is based on the use of a pulsed laser beam to transfer the hydrogel/bioink from a laser-beam-sensitive support to a flat collector [15,16,19]. EBB consists of an automated robotic system for extrusion of the hydrogel or bioink in the shape of cylindrical filaments that form the desired 3D structure [20,21]. The latter technique is the most widely investigated for the preparation of hydrogel scaffolds, which can be loaded with cells. There are several hydrogels that can be used in EBB; hydrogels can be based on natural or synthetic polymers and they can be used alone or blended with other hydrogels to improve cell/material interaction [20].

In this work, to the best of our knowledge, MC hydrogels were 3D printed for the first time in order to use them for the biofabrication of oriented CS. Accordingly, two MC-based hydrogels were prepared using the same MC concentration and different saline solutions and were applied with the aim of obtaining 3D-printed CSE substrates via extrusion-based bioprinting. After physical characterization, the resulting 3D-printed substrates were tested with N1H/3T3 murine fibroblasts and MS1 murine endothelial cells for the production of CS having ring-shaped geometry.

## 2. Materials and Methods

### 2.1. Materials

Methylcellulose (MC, Methocel A4M, g = 4000 mPa × s for a 2% *w*/*v* aqueous solution at 20 °C) was kindly supplied by The Dow Chemical Company (Midland, MI, USA). All basic chemicals were from Sigma-Aldrich (Saint Louis, MO, USA) unless stated otherwise.

### 2.2. Hydrogel Preparation

Methylcellulose hydrogels were prepared using a dispersion technique, as reported elsewhere [13,22]. Aqueous solutions were prepared using 0.05 M Na_2_SO_4_ solution (MC-Na005) or in 20 g/L phosphate buffered saline (PBS) (MC-PBS20). MC powder was added after heating the saline solutions to 55 °C to ensure a homogeneous powder dispersion. The resulting polymeric suspensions (Table 1) were then stored in a refrigerator at 4 °C for 24 h to allow complete hydration of the MC.

### 2.3. Rheological Analysis

The MC hydrogels were characterized rheologically with a rotational rheometer (AR-1500ex, TA Instruments, New Castle, DE, USA), using flat-plate geometry (diameter = 2 cm, working gap = 1 mm). A homemade isolation chamber in polymethyl methacrylate (Plasting srl, Segrate, Italy) was designed and fixed to the rheometer to prevent dehydration of the hydrogel under test.

For each MC hydrogel composition, tests were run using three specimens taken immediately after 24 h hydration at 4 °C. The linear viscoelastic region (LVR) was identified at 20 and at 37 °C by applying a strain sweep between 0.1 and 100%, frequency 1 Hz. To determine the LCST, temperature sweep tests were then performed between 10 and 60 °C, applying a temperature ramp of 2 °C/min, 0.5% strain (i.e., LVR obtained by strain sweep test), frequency 1 Hz. The LCST was identified for each hydrogel composition, as reported elsewhere [22], from storage modulus (G’) and complex viscosity (η*) curves. Briefly, the LCST was obtained as the intersection between the interpolant of the initial T range, characterized by a slight decrease of the parameter considered, and the interpolant of the subsequent T range, characterized by an increase of the parameter.

### 2.4. 3D Printing of MC-Based Hydrogels

**Figure 1:**
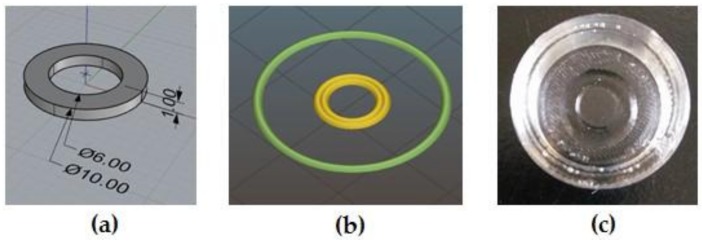
Printing process of the ring-shaped hydrogels in the polydimethylsiloxane (PDMS) support. (**a**) CAD model obtained using Rhinoceros software; (**b**) preparation of *.gcode* file; the control perimeter produced by Slic3r is shown in green; (**c**) Methylcellulose (MC)-based hydrogel printed in the PDMS support.

MC-based hydrogels were 3D-printed by an extrusion-based 3D printer (Kiwi 4D, Sharebot, Nibionno, Lecco, Italy) using a modified version of the printer filament specifically adapted to print hydrogels (Kiwi 3D printer, Sharebot, Nibionno, Lecco, Italy). Briefly, the Kiwi 4D printer allows the lodging of a 60 mL medical syringe into a cylindrical heating jacket, used to control the printing temperature depending on hydrogel characteristics. The extrusion process is controlled by a stepping motor connected to a piston that drives the plunger of the syringe. MC-specimens were 3D-printed directly on polydimethylsiloxane (PDMS, Elastosil^®^, Wacker, Munich, Germany) supports (Figure 1c); after the printing process was completed, the PDMS support and the MC hydrogel printed on it were lodged into a well of a tissue culture multiwell plate (12 wells TCPS, TCPS-12, (CORNING, Corning, NY, USA) for subsequent in vitro cultures. The PDMS supports were obtained via replica molding starting from poly-L-lactic molds obtained by PLLA filament extrusion (Sharebot Next Generation, Sharebot, Nibionno, Lecco, Italy). The procedure that was followed to obtain the printing files for the MC-hydrogels involved developing the desired geometry using 3D-modeling software, Rhinoceros (5.1, Robert McNeel & Associates, Seattle, WA, USA) (Figure 1a), converting the model into a *.stl* file and, lastly, running a slicing procedure with Slic3r (Figure 1b) to obtain the *.gcode* files. Considering that this was the first time that MC hydrogels had been 3D printed, an optimization of the hydrogel 3D printing process was required; accordingly, the optimal printing parameters for both the MC-hydrogel compositions were the following: T = 24−26 °C, 18 G needle, deposition speed = 1 mm/s, extrusion multiplier = 3. The latter parameter, corresponding to a descending speed of the piston (along the z-axis) of 1.45 μm/s, was chosen to achieve the deposition of a continuous strand of hydrogel.

Using the optimized parameters, ring-shaped specimens (internal diameter = 6 mm, external diameter = 10 mm) of MC-based hydrogels were obtained by printing one layer 1 mm in height.

### 2.5. Weight Variation Tests

The stability of the bulk (MC-PBS20-bulk and MC-Na005-bulk) and printed (MC-PBS20-print and MC-Na005-print) MC-based hydrogels was evaluated in distilled water at 37 °C for up to 7 days. The hydrogel specimens (*n* = 3) were weighed at the start of the test and incubated in 4 mL distilled water. At different time points (*t* = 1, 2, 3, 4, 7 days), excess water was removed, the weights of the specimens were recorded, and 4 mL of fresh distilled water was added. The weight variation (*ΔW*) was calculated as follows:

(1) $$\Delta W\%=\frac{w_{t}-w_{0}}{w_{0}}\times \;100$$

where $w_{t}$ is the specimen weight at time *t* and $w_{0}$ at time 0.

### 2.6. In Vitro Biological Tests

For the in vitro biological tests, two differently shaped specimens were tested: Bulk and print-ring specimens. MC-based hydrogels were prepared using a filter-sterilized solution (0.22 μm pore size filters, VWR International, Milan, Italy) and UV light sterilized MC powder. Bulk specimens (*n* = 3) were prepared by adding MC solutions in a tissue culture multiwell plate (24 wells TCPS, TCPS-24). The print-ring specimens were printed with the Kiwi 4D printer under a laminar flow cabinet; the printer was moved under the cabinet and disinfected with 70% *v*/*v* ethanol solution and sterilized by UV light for 30 min before MC printing. In the in vitro tests, murine embryonic fibroblasts (NIH/3T3, CRL-1658, ATCC, Manassas, VA, USA) modified with a gene for the expression of green fluorescent protein (GFP) and endothelial murine cells (MS1, CRL-2279, ATCC, Manassas, VA, USA) were selected to investigate the feasibility of using different cell phenotypes to produce CS. For both cell lines, Dulbecco Modified Eagle’s Medium (DMEM, Sigma-Aldrich, Saint Louis, MO, USA) supplemented with fetal bovine serum (FBS, 10% *v*/*v*), L-glutamine (1% *v*/*v*), Hepes (1% *v*/*v*), sodium pyruvate (1 mM, 1% *v*/*v*), and a solution of penicillin/streptomycin (1% *v*/*v*) was used.

#### 2.6.1. Cell Viability Tests

Previous in vitro cytotoxicity tests [13] had found that neither of the two types of hydrogel (Table 1) released low molecular weight substances with possible toxic effects on the cells. Before proceeding with CS production, cytocompatibility tests were performed, evaluating cell viability by the alamarBlue (alamarBlue^®^, Thermo Fisher, Waltham, MA, USA) assay. For all possible combinations of MC-based hydrogel (Table 1)/specimen type (bulk, print-ring)/cell phenotype (3T3, MS1), cell viability was assessed. Type I collagen extracted from rat tail (BD, 2 mg/mL) was used to coat each specimen in order to promote cell adhesion and proliferation [13]. Cells were then seeded (cell density = 1.5 × 10^5^ cells/well) onto each specimen and the culture plates were stored in an incubator (37 °C, 5% CO_2_) for 24 or 48 h. Tests were performed in triplicate. Cells cultured on TCPS alone at the same density were used as control.

#### 2.6.2. Cell Sheets

Following the procedure described above, MC hydrogel samples (bulk and printed specimens) were prepared for cell sheet detachment tests. After 48 h of culture in the incubator (37 °C, 5% CO_2_), the plates were removed and placed at 4 °C for 20 min. Once spontaneously detached from the hydrogel surface, cell sheets were sucked up with a sterile pipette and moved onto a fresh TCPS plate for subsequent characterization by immunofluorescence analysis. Tests were performed in triplicate. For all possible combinations of MC-based hydrogel/specimen shape/cell phenotype, a cell sheet specimen was used for immunofluorescence analysis. All detached cell sheets were transferred onto electrostatic slides and stained with phalloidin to stain the actin filaments and with 4′,6-diamidino-2-phenylindole (DAPI) to co-stain the nuclei. The specimens were then observed through a fluorescence microscope (Leica DM5500 B, Leica, Basel, Switzerland). Images were then processed (ImageJ, v. 1.51f, NIH, National Institutes of Health, USA) to quantify the mean circularity of the nuclei in each type of cell sheet. Circularity is expressed by mean of a numerical value between 0 and 1, where 1 meant a perfect circle and 0 a stretched polygon, as described by Altomare et al. [23].

For all possible combinations of MC-based hydrogel/specimen type/cell phenotype, CS detached from the MC hydrogels were transferred into a fresh multiwell plate (TCPS-12) in order to qualitatively evaluate cell sheet adhesion and, hence, cell proliferation on the new substrate. As described elsewhere [13], a few drops of medium were gently spotted directly onto the CS to avoid dehydration. Then, 150 μL of complete DMEM was added and, after 6 days of culture, plates were observed by fluorescence microscopy (Leica DM5500 B).

#### 2.6.3. Co-Cultured Cell Sheets

To test the possibility of obtaining cell sheets made of more than one cell population, MS1 (endothelial cells) and NIH-GFP^+^ (fibroblasts) cells were seeded onto 3D-printed supports filled with both MC-Na005 and MC-PBS20 hydrogels. Cells were seeded in a 50:50 ratio (1.5 × 10^5^ cells/support) and cultivated for 48 h. Co-cultured cell sheets were then detached by lowering the temperature as described above (Section 2.6.2), collected, and stained with an anti-alpha smooth muscle actin antibody (α-SMA, ab5694, from Abcam, Cambridge, UK) specific for endothelial cell cytoskeleton. Lastly, detached co-cultured cell sheets were co-stained with DAPI and images were collected using a fluorescence microscope (Leica DM5500 B).

#### 2.6.4. Cell Sheet Superimposition

To test the possibility of biofabricating complex multi-layered proto-tissues in vitro, CS made of (i) NIH-GFP^+^ fibroblasts and (ii) MS1 endothelial cells were produced separately onto 3D-printed molds and filled with either MC-Na005 or MC-PBS20 hydrogels. NIH-GFP and MS1 CS were then detached and superimposed to obtain a sandwich comprising three layers in the order NIH–MS1–NIH. After 48 h of direct layer-to-layer contact, three-layered cell sheets were stained with α-SMA, GFP, and DAPI to detect the presence and the interaction of the three layers.

### 2.7. Statistical Analysis

Experiments were performed using at least three replicates for each assay. Data have been expressed as the mean ± standard deviation and compared statistically by means of the two-sample t-test (significance level = 0.05; GraphPad PRISM, v.6.0, GraphPad Software, La Jolla, CA, USA. For nuclei circularity analysis, the non-parametric Mann–Whitney U-test was performed (significance level = 0.05, GraphPad PRISM, v.6.0), evaluating the normal distribution by the Shapiro–Wilke test.

## 3. Results and Discussion

### 3.1. Qualitative Analysis of MC-Based Hydrogels

**Figure 2:**
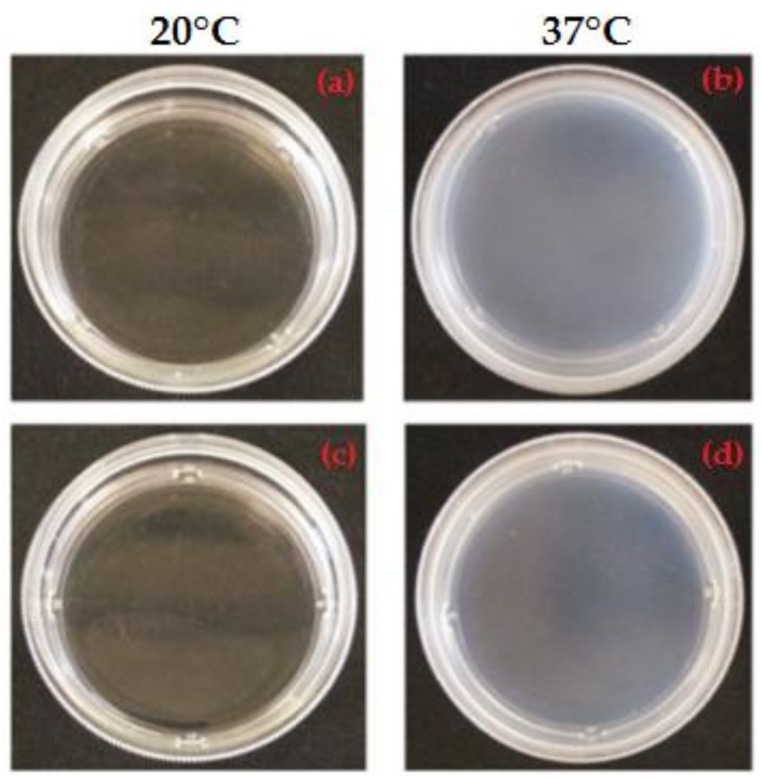
Sol-gel transition of the MC-based hydrogels tested: sol, hydrophilic state at 20 °C (T < LCST): (**a**) MC-Na005, (**c**) MC-PBS20; gel, hydrophobic state at 37 °C (T > LCST): (**b**) MC-Na005, (**d**) MC-PBS20.

Based on previous research [13,22], the MC-based hydrogels were selected for their ability to undergo a sol–gel transition upon temperature variation. Proper Na_2_SO_4_ and PBS concentrations were selected to achieve a salting-out effect, thus reducing the LCST of MC aqueous solutions to 37 °C, suitable for applications in the field of CSE [13,22]. LCST reduction for MC-Na005 and MC-PBS20 solutions is triggered by SO_4_^−^ ions (deriving from Na_2_SO_4_ dissociation) and Cl^−^ ions (deriving from KCl and NaCl dissociation), respectively, inducing water molecule spillage from the polymeric structure [13,22,24]. Macroscopic observation (Figure 1) show that both hydrogels appeared transparent at 20 °C (Figure 2a,c) whereas after being allowed to stabilize for 1 h at 37 °C, they became visibly opaque (Figure 2b,d). This indicates the successful transition of the hydrogels from the sol to gel state, as verified in previous work [13]. In particular, at 20 °C, the MC-based hydrogels are in the sol state and hydrogen bonds between the water molecules and the hydrophilic groups (–OH) of the MC chains predominate. Conversely, at 37 °C, the hydrophobic groups of MC (–OCH_3_) are exposed and associate hydrophobically to form a physically cross-linked structure [7,8,9,10]. The sol/gel transition of the hydrogel is accompanied by a change in its affinity for water, from a hydrophilic to a hydrophobic state. This process is reversible since the physical cross-linking does not involve the formation of covalent bonds. Inversion tests have been run to quantitatively investigate the LCST of various MC-based hydrogels [7,13,25,26]. However, the high concentration of MC used in the present study to prepare MC-Na005 and MC-PBS20 made these hydrogels too viscous to evaluate the sol/gel transition by the inversion test. Rheological analyses were performed to quantitatively evaluate the LCST values.

### 3.2. Rheological Analysis

**Figure 3:**
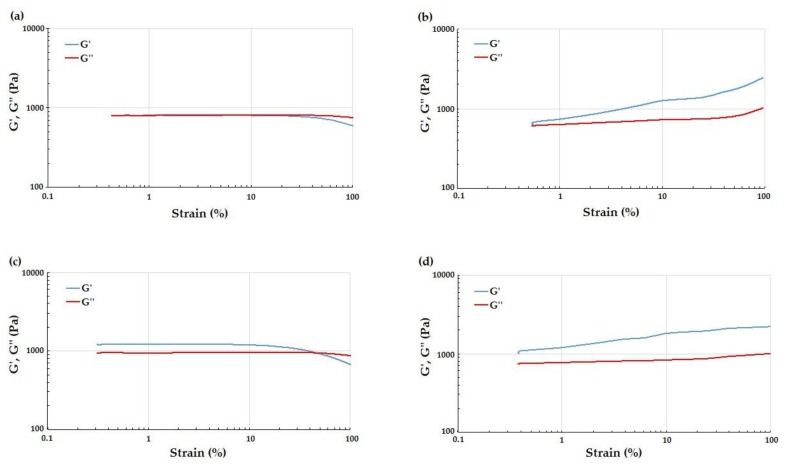
Representative graphs of G’ and G’’ in the strain sweep tests performed at 20 °C for (**a**) MC-Na005 and (**c**) MC-PBS20; and at 37 °C for (**b**) MC-Na005 and (**d**) MC-PBS20.

The LVR region of MC-based hydrogels was identified by means of strain sweep tests. The curves at 20 °C (Figure 3a) of both types of hydrogel showed a linear behavior of G’ and G’’ up to strain values close to 50%. At 20 °C, G’ and G’’ curves were almost coincident, although the loss modulus was expected to be higher than the conservative modulus for a hydrogel in the sol state [27,28]. This could have been caused by the fact that at 20 °C the hydrogel was already undergoing the sol-gel transition. For strain values above 50%, the hydrogel’s behavior was non-linear and the storage modulus decayed because of the break of the polymeric network structure (Figure 3a,c). At 37 °C (Figure 3b), the values of G’ remained higher than those found for G’’ in the entire strain range tested, indicating a predominant contribution of the elastic component versus the viscous component, typical of hydrogels in the gel state. In addition, G’ and G’’ curves lost linearity for deformation between 0.5% and 1%. Resulting from the strain sweep tests, the strain in the subsequent temperature sweep tests was set at 0.5% in order to ensure the linearity of G’ and G’’ for both the MC-based hydrogel formulations.

**Figure 4:**
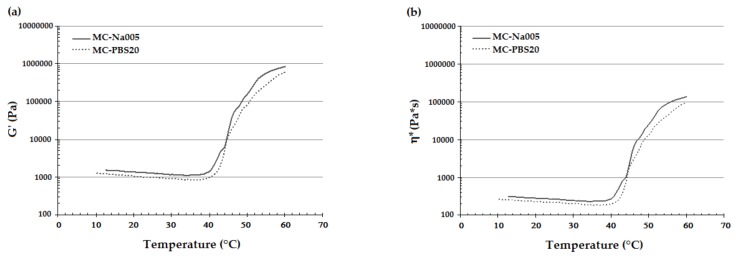
Representative trend of G’ and η* resulting from the temperature sweep tests performed on MC-Na005 and MC-PBS20 hydrogels: (**a**) G’/T curves, (**b**) η*/T curves.

The temperature sweep test produced a graph of G’ and η* as a function of temperature for each specimen (Figure 4). This trend is important for printing the hydrogel, enabling the appropriate temperature for good printability and shape fidelity to be set [29] and that for detaching the CS to be determined [2,3,8,13]. The curves of G’ (Figure 4a) and η* (Figure 4b) showed a similar trend for the two considered MC hydrogel formulations and are in accordance to that reported in the literature for MC-based hydrogels from other authors [30,31]. In particular, there was an initial decrease in both viscoelastic parameters due to disentanglement of the polymer chains. On increasing the temperature, the hydrogen bonds between the water and –OH groups of the MC break due to the transition from the sol to the gel state. Exposure of the –CH_3_ groups of the MC thus leads to the association of the hydrophobic polymer chains of MC [13,30,31]. In response to this phenomenon, G’ and η* undergo a rapid increase. Further, the curves of G’ and η* also showed a change in the curve’s concavity, indicating that the hydrogel had reached the *plateau*. The LCST values were obtained from the G’/T and η*/T curves (Table 2) as described above. No significant difference was found in the LCST value obtained from the G’/T and η*/T curves, and that from the MC-Na005 and MC-PBS20 hydrogels. The LCST values enabled the temperature for the printing process to be set in the temperature range 24–26 °C as at these temperatures the rheological properties of the two hydrogel formulations are stable and the MC-based hydrogels can be extruded by the printer needle.

### 3.3. 3D Printing of MC-Based Hydrogels

**Figure 5:**
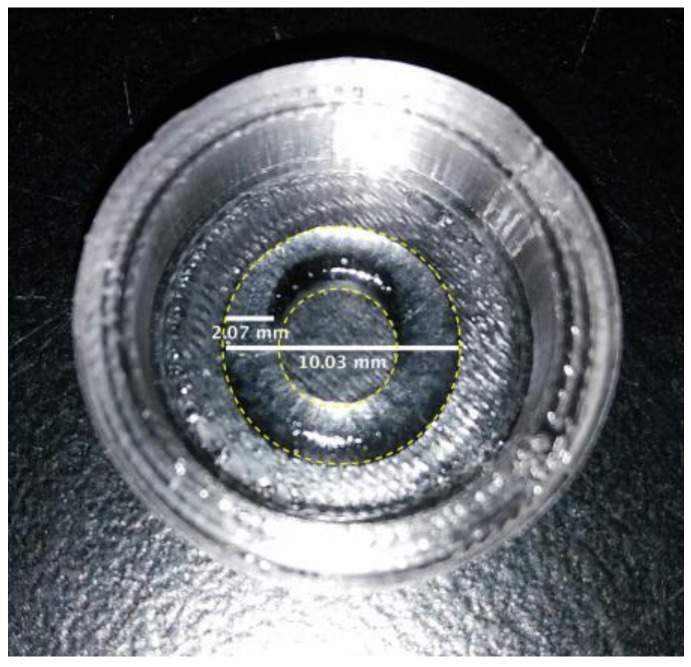
Representative printed ring in MC-Na005 hydrogel in the M12 support. The inner and outer diameters are shown by dotted lines.

After parameter optimization, due to the novelty of the technique, MC hydrogel rings were successfully printed with both MC-based formulations within the PDMS supports. A representative image of the hydrogel printed ring is shown in Figure 5; the dimensions were not significantly different from the expected values (φ_int_ = 6 mm, φ_ext_ = 10 mm). To the authors’ knowledge, this is the first time that MC-based hydrogels have been printed. In other studies reported in the literature, MC has been blended with other natural polymers for the printing process, rather than for the production of CS. Schutz et al. [32] reported printing MC and alginate to increase the viscosity of the alginate, permitting printing at room temperature. The use of MC led to enhanced viscosity, improving the alginate printing conditions. MC can be released from the printed scaffolds during the subsequent in vitro cell cultivation. Other studies have reported the use of MC in blends to print drug-release systems [33] or to improve adhesion between the printed layers [34].

### 3.4. Weight Variation Tests

**Figure 6:**
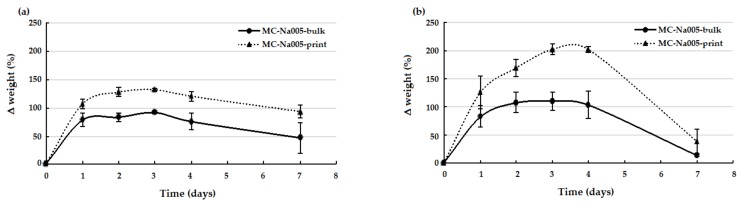
Weight variation (*ΔW*%) versus incubation time in the stability tests performed in distilled water on bulk and printed MC-based hydrogels: (**a**) MC-Na005; (**b**) MC-PBS20.

The weight variation trend over time (up to 7 days) was similar for the two MC hydrogel formulations analyzed, both before and after printing (Figure 6). In particular, after rapid initial swelling, all samples reached a plateau, which was maintained until day 4, except for the MC-PBS20 printed hydrogel, which continued to swell until day 4 (Figure 6b). After 4 days of incubation, for all samples, a weight decrease occurred, indicating the start of the degradation process. An analysis of the weight variation for the bulk and print specimens showed that for the MC-Na005 hydrogel formulation (Figure 6a), the printing process determined a significant increase (*p* < 0.05) in weight variation during swelling (days 1–4), following a similar trend for the two types of specimens. In the case of the MC-PBS20 hydrogel (Figure 6b), there was a significant difference between the printed and the bulk hydrogel throughout the test period (*p* < 0.05). The larger weight variation of the MC-based printed hydrogels may have been caused by the shear stress induced by the printer needle on the gel during the printing process. As a consequence of extrusion, the MC chains in the printed hydrogel align and approach so closely to one another that the effect is comparable to an increase in concentration of MC in the gel: An increase in MC concentration determines an increased swelling of MC-based hydrogels, as reported by Thirumala et al. [8].

### 3.5. In Vitro Biological Tests

#### 3.5.1. Cell Viability Tests

**Figure 7:**
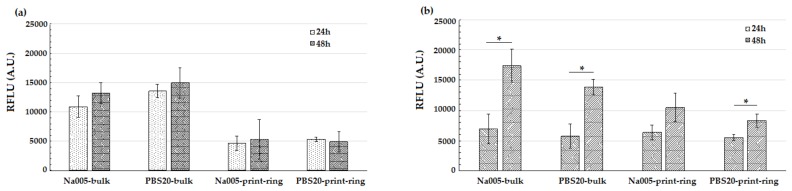
alamarBlue assay on MC-Na005 and MC-PBS hydrogel, bulk and printed ring specimens; cells were cultured on the specimens for 24 and 48 h. (**a**) NIH/3T3 fibroblast cells; (**b**) MS1 endothelial cells. (*): *p* < 0.05, comparing 24 versus 48 h of culture.

The viability of murine fibroblasts and murine endothelial cells was evaluated after 24 and 48 h in culture on the MC-based hydrogels under study. For bulk and printed ring specimens, the alamarBlue assay showed increased fluorescence values (Figure 7) between 24 and 48 h after seeding, indicating proliferation and good viability of cells in contact with the MC-based hydrogels. In particular, specimens seeded with NIH/3T3 murine fibroblasts (Figure 7a) showed viability values that did not significantly differ at 24 and 48 h. Conversely, for MS1 endothelial cells (Figure 7b), the increase in cell viability differed significantly (*p* < 0.05) for MC-Na005 bulk and MC-PBS20 bulk and printed ring specimens. Thus, the 3D printing technique did not affect cell viability of the resulting CS. Comparing the two hydrogels (MC-Na005 versus MC-PBS20), no significant differences emerged between the two formulations either for bulk or for printed ring specimens.

#### 3.5.2. Characterization of Cell Sheets

**Figure 8:**
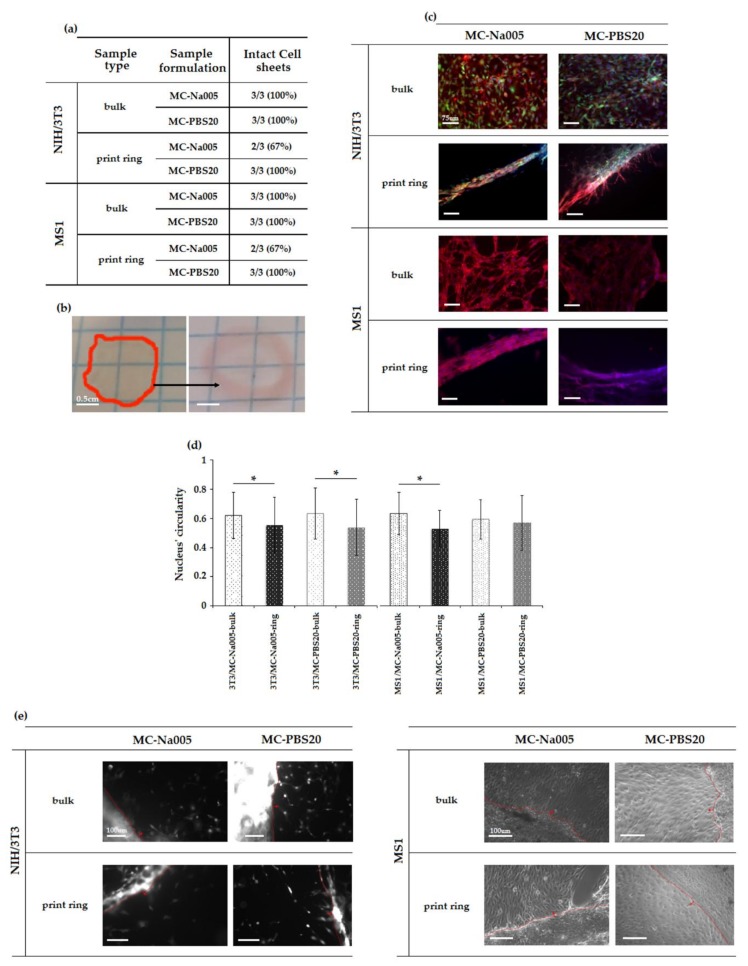
Characterization of the cell sheets (CS) obtained by culturing NIH/3T3 and MS1 cell phenotypes on the MC-based hydrogel formulations under study (MC-Na005 and MC-PBS) for the two specimen types (bulk and printed ring). (**a**) Efficiency of CS detachment. (**b**) Representative macro image of detached CS: left) NIH/3T3 CS detached from MC-PBS20 bulk hydrogel, right) MS1 CS detached from MC-PBS20 printed ring hydrogel. Scale bar = 0.5 cm. (**c**) Fluorescence images of NIH/3T3 and MS1 CS detached from MC-Na005 and MC-PBS20 hydrogels in bulk and printed ring shape. (**d**) Circularity of nuclei of the CS obtained for the different combinations of MC-based hydrogel/specimen type/cell phenotype. (**e**) Fluorescence and optical microscopy images of CS cultured for 6 days in a fresh TCPS; cell migration from the CS to the well bottom can be observed.

For all combinations of MC-based hydrogel formulation/specimen type/cell phenotype, CS were detached after 48 h of culture. Complete and ready detachment of CS from the surface of the hydrogels was achieved by placing the culture plates at 4 °C for 20 min. With a decrease in temperature, as was said above, the structure of the MC-based hydrogels changed to a hydrophilic sol state. Thus, the cells spontaneously detached from the hydrogel without the need for proteolytic enzymes (e.g., trypsin). For each type of sample, the efficiency of the detachment operation has been expressed as the number of intact detached cell sheets versus the total number of seeded hydrogels (Figure 8a). All CS detached intact from either bulk or printed ring specimens but the detachment efficiency for the printed specimens was lower than for the bulk specimens. For the same type of specimen (bulk or printed ring), no macroscopic difference emerged in CS between the two compositions (MC-Na005 and MC-PBS20) nor between the two cell phenotypes (NIH/3T3 and MS1). CS detached from the bulk hydrogel (Figure 8b, left) exhibited a round shape with a diameter below 10 mm (TCPS-24 well diameter = 15.6 mm) due to shrinkage of the CS after detachment, confirming results reported in the literature [35,36]. Conversely, CS detached from the printed ring hydrogels (Figure 8b, right) showing sizes in agreement with the expected geometry. This result, together with observations concerning the efficiency of the detachment procedure, confirmed that the 3D printing technique is adequate for obtaining ring-shaped cell sheets, and it could be promising for obtaining other complex geometries.

Fluorescence images acquired for GFP (green, only for NIH/3T3 fibroblasts), phalloidin (red), and DAPI (blue) staining highlight the cell organization on the bulk and the printed ring MC-based hydrogels. CS produced on bulk hydrogels (Figure 8c) were compact and qualitatively the cells appeared cohesive. Conversely, CS on the printed ring hydrogels showed the expected ring shape (Figure 8c). No qualitatively-different cell morphology appeared between the images acquired for the two MC-based hydrogel formulations. In the images obtained by immunofluorescence staining, the nuclei (in blue, Figure 8c) were surrounded by an extensive red region, indicating the actin filaments constituting the cytoskeleton and ensuring cell junctions in the resulting sheet. Given the continuity between the actin filaments, a structure similar to a cell layer appeared to have formed. With regard to the ring-shaped CS, at an initial qualitative observation, the cells exhibited a preferential orientation, as shown by the directionality of the cell cytoskeleton. Therefore, the cell orientation was obtained thanks to the printed geometry that influenced the cytoskeleton spread elongation.

The influence of the printed geometry towards cells orientation was further confirmed by the elongated shape taken on by the cell nuclei, for both phenotypes studied. In fact, analysis of the DAPI stained fluorescence image (i.e., nucleus staining), aimed at quantifying the circularity of cell nuclei present on each cell sheet, showed that the mean circularity values for bulk sheets were close to 1 (indicating perfect circularity) whereas those for the printed ring differed significantly (*p* < 0.05), except in the case of the MC-PBS20 hydrogels cultured with MS1 endothelial cells (Figure 8d). It may thus be assumed that the seeded cells adhered to the hydrogel surface and proliferated following the ring shape of the printed hydrogel.

Having produced the cell sheet, three sheets for each combination of MC-based hydrogel/specimen type/cell phenotype were transferred to a fresh 12-well TCPS to investigate cell sheet adhesion and colonization on a new substrate. Qualitative fluorescence microscopy analysis was used for NIH/3T3 CS and optical microscopy analysis for MS1 CS. After 6 days of culture, cells of all sheets began to migrate from the edge of the sheet (dotted line, Figure 8e) and proliferate on the bottom of the TCPS well. Cells in the CS appeared closely interconnected with one another since the gap junctions had not been destroyed when the cells were detached from the MC-based hydrogel. These results are encouraging in view of applications in the field of regenerative medicine. The sheets, preserving their cell junctions, can be used as a tissue patch and introduced by a mini-invasive approach to the damaged site. The advantage of this technique is in the possibility to implant CS at the defect site without the use of sutures, exploiting the natural adhesion of the ECM proteins.

#### 3.5.3. Co-Cultured and Three-Layer Cell Sheet Analysis

**Figure 9:**
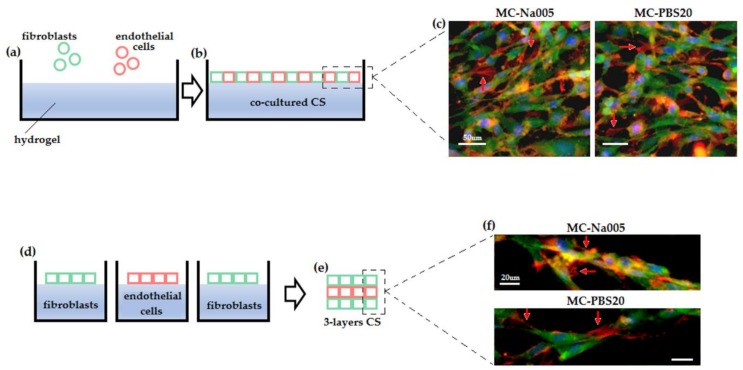
Co-cultured and three-layer CS. When MS1 endothelial cells and NIH-GFP^+^ fibroblasts were co-cultured to obtain multi-species CS (**a**,**b**), both Na005 and PBS20 MC hydrogels were able to support the presence of both cell populations: both green and red signals could be detected from GFP (NIH) and α-SMA (MS1, highlighted by the red arrows), together with the blue stain for the nuclei (**c**). Moreover, when three single-cell CS were collected and superimposed to obtain a three-layer sandwich (**d**,**e**), again green and red signals sometimes overlapped, suggesting an active interaction between the cells of the different layers (**f**).

The final step of the cell sheet biofabrication investigation investigated the possibility of obtaining (i) multi-species and (ii) multi-layer CS. The results are shown in Figure 9.

To obtain multi-species CS, endothelial cells (MS1) and fibroblasts (NIH-GFP^+^) were seeded together, mixing them in a 50:50 ratio to avoid any predominant population (Figure 9a,b). To obtain the MS1–NIH CS, the same procedure as for a single-species cell sheet was followed: cells were seeded at high confluence (1.5 × 10^5^/support) on a collagen coating and detached after 48 h by cooling the system, affording hydrogel phase transition. As shown in Figure 9c, both hydrogel compositions (Na005 and PBS20) successfully supported the formation and detachment of MS1–NIH CS: IF staining evidenced the presence of both cell populations in the detached CS. Endothelial cells were detected by the red signal from the targeted anti-alpha smooth muscle actin antibody, while fibroblasts were stained in green (auto-GFP signal). The cells were shown to interact with one another, forming a continuous and homogeneous layer of fully interconnected closely packed cells.

The possibility of interactions occurring between detached CS of different origins was also investigated. MS1 and NIH-GFP^+^ CS were produced separately on 3D-printed supports, detached by cooling the hydrogel, and collected. Successively, using MS1 and NIH CS, a three-layer sandwich was produced by superimposing CS made of NIH–MS1–NIH cells, respectively (Figure 9d,e). The results are very encouraging since the three layers successfully interacted with one another to form a complex in vitro biofabricated tissue, in which all three layers were detectable. As shown in Figure 9f, in both Na005 and PBS20 hydrogels two layers of fibroblasts were visible (stained in green) interspersed by a red-stained layer of endothelial cells (MS1), again detected by the specific anti-α-SMA antibody. Interestingly, the three layers did not appear as sharply separated layers. Instead, they showed a certain interaction with each other with the green and red signals overlapping. This suggests cross-talk involving the cells of the different layers.

## 4. Conclusions

Two MC-based hydrogels were characterized for their possible application in the field of CS engineering. The possibility of printing MC-based hydrogels by an extrusion-based printing technology was investigated for the first time and successfully applied. The resulting CS showed that the 3D printing technique is a promising strategy to obtain CS that replicate the geometry of the printed MC hydrogel substrate. This allows the acquisition of more complex-shaped CS, compared to those obtained by culturing cells on MC hydrogels poured into simple-shaped molds. The cell orientation in the ring-shaped CS, confirmed by the degree of circularity of the nuclei, was of particular interest.

## Figures and Tables

**Table 1:** Composition of the MC-based hydrogels.

Sample	MC Concentration (%, *w*/*v*)	Salt Type	Salt Concentration
MC-Na005	8%	Na_2_SO_4_	0.05 M
MC-PBS20	8%	PBS	20 g/L

**Table 2:** Values of the sol/gel transition temperature (lower critical solution temperature (LCST)) for MC-Na005 and MC-PBS20. The sol/gel transition temperature was determined by the temperature sweep tests.

Sample	LCST [°C] from G’/T Curve	LCST [°C] from η*/T Curve
MC-Na005	41.3 ± 1.1	41.7 ± 1.2
MC-PBS20	39.4 ± 3.1	39.7 ± 3.1
